# Effect of maternal glycemia and weight status on offspring birth measures and BMI-z among Chinese population in the first year

**DOI:** 10.1038/s41598-017-15932-2

**Published:** 2017-11-22

**Authors:** Yilin Huang, Baoming Yin, Xiaohong Liang, Hong Mei, Hongyan Lu, Shuixian Xie, Weihong Bei, Wenhua Mei, Jianduan Zhang

**Affiliations:** 1 Department of Maternal and Child Health Care, School of Public Health, Tongji Medical College, Huazhong University of Science and Technology, Hangkong Rd., Wuhan, 430030 Hubei China; 2 Department of Gynecology and Obstetric, Maternal and Child Health Hospital of Zhuhai Municipality,, 543 Ningxi Rd., Zhuhai, 519001 Guangdong China; 3 Department of Child Health, Maternal and Child Health Hospital of Zhuhai Municipality,, 543 Ningxi Rd., Zhuhai, 519001 Guangdong China; 4 Public Hospital Administration of Zhuhai Municipality,, 41 Jiaoyu Rd., Zhuhai, 519000 Guangdong China

## Abstract

To investigate the effects of maternal fasting plasma glucose (FPG) and pre-pregnancy weight status (PPWS) on offspring birth measures and body mass index z-score (BMI-z) in the first year, we conducted a prospective study of 1,096 mother-infant dyads in Guangdong, China, 2014–2015. Multivariate logistic regression was used to test independent/interaction associations of maternal FPG and PPWS with macrosomia/large for gestational age (LGA). Association of PPWS and FPG with offspring BMI-z in the first year was assessed by the linear mixed effects models. For each 1-mmol/L increase in FPG, the risk of macrosomia and LGA was elevated by 2.74 and 2.01 (95% CI: 1.85, 7.60 and 1.54, 5.88), respectively. No main effect of PPWS or interaction association of FPG and PPWS on macrosomia/LGA was observed (*P* > 0.05). A relation between maternal FPG and PPWS was detected (*P* < 0.05). Infants of Q5 FPG mothers, those who were born to OWO mothers, had a 0.35 increase in the BMI-z (95% CI: 0.16, 0.55) compared with infants of NW mothers. In conclusion, maternal FPG is positively associated with macrosomia/LGA. Maternal PPWS and FPG considerably interacted for the association with the risk of offspring high BMI-z in the first year.

## Introduction

The epidemic of childhood obesity has become an urgent public health priority due to its short and long-term development of diseases^1^ and economic burdens^2^. Infancy is the period during which children experience most rapid growth and can play a vital role in the later childhood obesity^3,4^. According to the Report on Chinese Residents’ Nutrition and Chronic Disease Status in 2015, the prevalence of overweight and obesity among children 0–6 years of age was 8.4% and 3.1%, in 2013, compared with 6.5% and 2.7%, in 2002, respectively. Accordingly, identifying risk factors related to excessive adiposity in children is of great importance for early obesity intervention. Several early life factors, such as maternal pre-pregnancy weight status (PPWS), intrauterine nutrition status (i.e. maternal hyperglycemia environment), woman age, gestational week, feeding pattern, etc., have been identified to be associated with weight status in childhood^5–12^.

GDM, which is defined as glucose intolerance with an onset or first recognition during pregnancy, is found to be a common pregnancy complication affecting around 1–28% of pregnancies according to a survey in 173 countries^13,14^. A Chinese study reported that between January 2016 and July 2016, 21.8% pregnant women were diagnosed with GDM based upon the criteria of the International Association of Diabetes and Pregnancy Study Groups (IADPSG) in some areas^15^. Most studies have investigated the relationship between GDM and gestational outcome^16,17^, which suggested that infants born to GDM mothers had higher weight or greater risk of large for gestational age (LGA). However, there exist some problems in the previous studies. First, different diagnostic criteria were used in these studies, which has led to incomparable results. Second, simply splitting pregnant women into GDM and non-GDM might neglect those who are at the higher end of hyperglycemia level but do not yet meet the diagnosis criteria. A recent study highlighted that higher maternal FPG concentration, which is commonly used in the diagnosis of diabetes and is usually performed in the morning and requires people to avoid food consumption for at least 8 hours before being tested, not necessarily GDM, was already significantly associated with greater offspring birth size and later childhood obesity^18,19^. Other studies suggested that detection and management of mild hyperglycemia below the current diagnostic criteria of GDM were necessary for improving pregnancy outcomes. The strength of the conclusion was limited by the comparatively small sample size (n = 258)^20^, accordingly further large-scale studies are needed.

Maternal pre-pregnancy obesity is another key predictor of offspring weight status. Leng *et al*.^8^ found that women who were overweight/obese (OWO) before pregnancy or had excessive gestational weight gain (GWG) could have an increased risk of having an infant with LGA/macrosomia and overweight from age 1–5 years. Aris *et al*.^9^ conducted a study with 2,853 Polish patients from 56 diabetes outpatient clinics and found that LGA and macrosomia had positive correlation with maternal pre-pregnancy weight. Independent of GDM, maternal obesity may also be characterized by insulin resistance and elevated lipids, allowing excess nutrients to be transferred across the placenta to the fetus, thus contributing to fetal growth^21^. Maternal pre-pregnancy obesity and GDM are intertwined^22^, but the influence of maternal gestational glycemia and maternal pre-pregnancy obesity have been studied separately, and it is still unclear whether maternal gestational glycemia plays an independent role or has the stacking effect together with maternal pre-pregnancy obesity on gestational outcomes and offspring BMI in childhood^23,24^. Instead of using maternal weigh status, some of the previous studies have also explored the effect of the association between maternal BMI and gestational glycemia on macrosomia/LGA. For example, a study by Liu *et al*. examined the impact of maternal gestational glycemia and BMI on macrosomia and found that high BMI, measured at GDM screening, was the most important determinant for risk of macrosomia^25^. However, some important confounders, like maternal GWG, were not considered, which might to some extent compromise the reliability of the conclusion. The study focused on population from Northern China, which might also affect the generalizability of its conclusions. A study by Heude *et al*. explored the role of pre-pregnancy BMI and net GWG on the risk of LGA in the French population, after adjusting for some key confounders. They found that higher net GWG was significantly associated with an increased risk of LGA only after accounting for blood pressure and glucose metabolism disorders. However, due to the difference in ethnicity, life styles and many other characteristics between Chinese and French populations, the conclusion drawn from the results of a study performed in the French population might not be generalizable to the Chinese population^26^. Furthermore, few epidemiological studies have taken into account factors like feeding pattern which are significant confounders regarding offspring’s overweight/obesity^17,20,27,28^.

Accordingly, the aim of this prospective study is to examine the independent and interaction effects of maternal PPWS and gestational glycemia on offspring birth measures and body mass index z-score (BMI-z) during the first year of life (i.e., at birth, 1, 3, 6 and 12 months) after adjustment for established confounders, such as maternal factors (i.e. woman age at delivery, GWG and gestational week) and feeding pattern.

## Results

### Characteristics of the study participants

The characteristics of the study participants based on the maternal FPG groups are listed in Table 1. The FPG concentrations were classified into 5 levels, namely the FPG quintile 1 group (mean: 3.9 mmol/l, n = 217, 19.8%), the FPG quintile 2 group (mean: 4.3 mmol/l, n = 219, 20.0%), the FPG quintile 3 group (mean: 4.5 mmol/l, n = 216, 19.7%), the FPG quintile 4 group (mean: 4.8 mmol/l, n = 209, 19.1%) and the FPG quintile 5 group (mean: 5.2 mmol/l, n = 235, 21.4%) according to the number of plasma glucose values of the oral glucose tolerance test (OGTT). In females, the range of gestational glycemia differed in FPG concentration, education, age, PPWS, primipara rate and delivery mode. In males, education performed different in the level of maternal gestational glycemia, and in offspring, the range of maternal gestational glycemia was different in the feeding pattern in the first month. The non-response analysis is shown in Table 2, it indicated a random lost to follow-up since no significant difference was found between those with full follow-up measures and those without, except for a comparatively lower paternal smoking rate.

**Table 1:** Characteristics of the study population according to quintiles of maternal FPG during pregnancy (n = 1096).

	Overall (n = 1096)	Q1 (n = 217)	Q2 (n = 219)	Q3 (n = 216)	Q4 (n = 209)	Q5 (n = 235)	F value	χ^2^	P value^1^
**Maternal characteristics**									
FPG, mmol/L	4.6 ± 0.5	3.9 ± 0.2^a2^	4.3 ± 0.1^b^	4.5 ± 0.1^c^	4.8 ± 0.1^d^	5.2 ± 0.3^e^	1340.7		<0.001
Education								22.9	0.03
Middle school and under	62(7.3)	12(7.1)^a^	16(9.0)^ab^	11(6.3)^ac^	9(5.4)^abcd^	14(7.6)^abde^			
High school	172(20.3)	42(24.7)	34(19.2)	26(14.8)	35(20.8)	35(19.0)			
College	222(26.3)	50(29.4)	46(26.0)	40(22.7)	63(37.5)	52(28.3)			
University above	390(46.1)	66(38.8)	81(45.8)	99(56.2)	61(36.3)	83(45.1)			
Age, y	28.7 ± 3.7	28.1 ± 3.7^a^	28.2 ± 3.4^ab^	28.2 ± 3.3^abc^	29.0 ± 4.0^abcd^	30.0 ± 3.8^e^	11.7		<0.001
PPWS, n(%)								36.1	<0.001
UW	253(23.2)	68(31.6)^a^	53(24.3)^ab^	48(22.2)^abc^	49(23.6)^abcd^	35(15.0)^ce^			
NW	725(66.5)	130(60.5)	152(69.7)	149(69.0)	139(66.8)	155(66.5)			
OWO	112(10.3)	17(7.9)	13(6.0)	19(8.8)	20(9.6)	43(18.5)			
Primipara, n(%)	890(81.2)	184(84.8)^a^	186(84.9)^ab^	180(83.3)^abc^	161(77.0)^abcd^	179(76.2)^abcde^		10.7	0.03
Delivery mode								42.4	<0.001
Natural	700(64.2)	159(73.6)^a^	153(70.5)^ab^	152(71.0)^abc^	120(57.4)^d^	116(49.6)^de^			
Cesarean	390(35.8)	57(26.4)	64(29.5)	62(29.0)	89(42.6)	118(50.4)			
Smoking, n(%)	31(2.8)	7(3.2)	10(4.6)	4(1.9)	6(2.9)	4(1.7)		4.3	0.37
Second hand smoking, n(%)	229(20.9)	38(17.5)	53(24.2)	45(20.8)	41(19.6)	52(22.1)		3.5	0.48
**Paternal characteristics**									
Education								22.8	0.03
Middle school and under	46(5.3)	6(3.6)^a^	12(6.8)^ab^	9(5.3)^bc^	8(4.9)^abd^	11(6.1)^abcde^			
High school	140(16.2)	36(21.3)	22(12.4)	18(10.5)	38(23.2)	26(14.3)			
College	222(25.8)	45(26.6)	50(28.3)	36(21.0)	44(26.8)	47(26.0)			
University above	454(52.7)	82(48.5)	93(52.5)	108(63.2)	74(45.1)	97(53.6)			
**Offspring characteristics**									
Male, n(%)	567(51.7)	106(48.8)	111(50.7)	113(52.3)	106(50.7)	131(55.7)		2.4	0.65
Gestational week	38.7 ± 1.4	38.8 ± 1.4	38.8 ± 1.4	38.7 ± 1.3	38.7 ± 1.4	38.7 ± 1.3	0.2		0.84
Birth weight, g	3158.7 ± 428.8	3128.0 ± 428.6	3185.9 ± 426.0	3141.9 ± 419.1	3173.3 ± 418.6	3164.4 ± 449.9	0.7		0.48
Birth length, cm	49.7 ± 1.3	49.6 ± 1.4	49.8 ± 1.4	49.8 ± 1.1	49.8 ± 1.4	49.8 ± 1.3	1.4		0.27
Macrosomia, n(%)	26(2.4)	4(1.8)	4(1.8)	5(2.3)	5(2.4)	8(3.4)		1.6	0.80
LGA, n(%)	29(2.6)	4(1.8)	4(1.8)	7(3.2)	5(2.4)	9(3.8)		2.7	0.60
Feeding pattern at 1 m, n(%)								16.5	0.04
EBF	310(45.1)	77(54.2)^a^	59(44.0)^ab^	52(40.0)^abc^	59(44.7)^abcd^	63(42.3)^bcde^			
MF	327(47.6)	60(42.3)	68(50.8)	70(53.8)	56(42.4)	73(49.0)			
FF	50(7.3)	5(3.5)	7(5.2)	8(6.2)	17(12.9)	13(8.7)			

^1^ *p* values denote differences across quartiles of FPG determined by ANOVA (parametric) or the Kruskal-Wallis test (nonparametric) for continuous variables and by the χ^2^ test for categorical variable. ^2^Significant difference of paired comparison across quartiles of FPG concentrations is represented as different letters of a–e. FPG, fasting plasma glucose; PPWS: pre-pregnancy weight status; UW: underweight; NW: normal weight; OWO: overweight/obese; LGA: large for gestational age; EBF, exclusive breast feeding; MF, mixed feeding; FF, formula feeding.

**Table 2:** Characteristics comparison between follow-ups and overall population.

	Missing value, n(%)	Overall (n = 1096)	Follow-ups (n = 822)	*t* value	χ^2^	*P* value^1^
**Maternal characteristics**						
FPG, mmol/L	0	4.6 ± 0.5	4.6 ± 0.5	0.03		0.96
Cars, n(%)	16(1.5)	606(56.1)	466(57.3)		0.28	0.60
Family monthly income, yuan	189(17.2)				4.61	0.47
≤3000		35(3.9)	27(4.0)			
3001–5000		143(15.8)	113(16.7)			
5001–10000		284(31.3)	182(26.9)			
10001–20000		317(35.0)	241(35.7)			
≥20001		128(14.0)	113(16.7)			
Education	221(20.2)				0.31	0.96
Middle school and under		62(7.1)	51(6.7)			
High school		172(19.6)	156(20.5)			
College		251(28.7)	213(28.0)			
University above		390(44.6)	341(44.8)			
Age, y	0	28.7 ± 3.7	28.8 ± 3.7	0.81		0.72
Pre-pregnancy BMI, kg/m^2^	6(0.5)	20.5 ± 2.6	20.6 ± 2.6	0.58		0.68
PPWS, n(%)	6(0.5)				0.25	0.88
UW		253(23.2)	192(23.4)			
NW		725(66.5)	540(65.7)			
OWO		112(10.3)	90(10.9)			
Primipara, n(%)	0	890(81.2)	674(82.0)			0.66
Gestational age at OGTT, week	0	25.4 ± 2.3	25.3 ± 2.3	−0.51		0.78
GWG, kg	30(2.7)	14.7 ± 4.1	14.3 ± 4.0	2.24		0.06
Delivery mode	6(0.5)				0.10	0.75
Natural		700(64.2)	533(64.9)			
Cesarean		390(35.8)	288(35.1)			
Smoking, n(%)	6(0.5)	31(2.8)	22(2.7)		0.04	0.84
Drinking, n(%)	5(0.5)	57(5.2)	46(5.6)		0.15	0.70
**Paternal characteristics**						
Weight, kg	48(4.4)	70.6 ± 10.6	70.9 ± 10.6	0.76		0.42
Height, cm	45(4.1)	172.5 ± 5.5	172.6 ± 5.5	0.37		0.68
BMI, kg/m^2^	48(4.4)	23.7 ± 3.1	23.8 ± 3.1	0.69		0.49
Education	234(21.4)				0.54	0.91
Middle school and under		46(5.3)	36(4.8)			
High school		140(16.2)	128(17.1)			
College		222(25.8)	186(24.9)			
University above		454(52.7)	398(53.2)			
Smoking, n(%)	44(4.0)	393(37.4)	249(32.0)		5.70	0.02
Drinking, n(%)	53(4.8)	328(31.4)	242(31.3)		0.00	0.95
**Offspring characteristics**						
Male, n(%)	0	567(51.7)	423(51.5)		0.01	0.91
Gestational week	0	38.7 ± 1.4	38.8 ± 1.3	1.11		0.55
Birth weight, g	0	3158.7 ± 428.8	3179.8 ± 428.8	1.07		0.40
Birth length, cm	0	49.7 ± 1.3	49.8 ± 1.2	1.09		0.43
Macrosomia, n(%)	0	26(2,0.4)	23(2.9)		0.18	0.67
LGA, n(%)	0	25(2.3)	22(2.7)		0.31	0.58
Feeding pattern at 1 m, n(%)	0				0.00	1.00
EBF		310(45.1)	310(45.1)			
MF		327(47.6)	327(47.6)			
FF		50(7.3)	50(7.3)			

^1^ *P* values denote differences between follow-ups and overall population determined by ANOVA (parametric) or the Kruskal-Wallis test (nonparametric) for continuous variables and by the*χ* ^2^ test for categorical variables. FPG: fasting plasma glucose; OGTT: oral-glucose-tolerance test; GDM: gestational diabetes mellitus; PPWS: Pre-pregnancy weight status; UW: underweight; NW: normal weight; OWO: overweight/obese; GWG: gestational weight gain; LBW: low birth weight; LGA: large for gestational age; EBF: exclusive breast feeding; MF: mixed feeding; FF: formula feeding.

### Effects of maternal glycemia and PPWS on offspring macrosomia/LGA at birth

For each 1-mmol/L increase in maternal FPG, the risk of offspring macrosomia and LGA at birth was elevated by 2.74 (95% CI: 1.85, 7.60; *P* = 0.00), and 2.01 (95% CI: 1.54, 5.88; *P* = 0.00), respectively, after adjustment for confounders like woman age at delivery, GWG and gestational week (Model 1 in Table 3). The association remained significant, the risk of offspring macrosomia and LGA at birth were elevated by 2.31 (95% CI: 1.62, 6.79; *P* = 0.00), and 1.69 (95% CI: 1.35, 5.33; *P* = 0.01) for each 1-mmol/L increase in maternal FPG, after further including maternal PPWS (Model 3 in Table 3). In the same model, the risk of offspring macrosomia at birth were elevated by 1.51 (95% CI: 0.89, 7.05; *P* = 0.08) for each 1-mmol increase in maternal FPG with marginal significance (Model 3 in Table 3), while in the model excluding maternal FPG, the association was more pronounced, i.e., mothers who were overweight/obese (OWO) before pregnancy were 3.14 times as likely to have an infant with macrosomia compared with normal weight (NW) mothers (95% CI: 1.14, 8.66; *P* = 0.03), (Model 2 in Table 3). No significant interaction effect of the maternal weight status and gestational glycemia on infants’ macrosomia/LGA was observed (Model 4 in Tables 3), (*P* > 0.05).

**Table 3:** Logistic regression RR (95% CIs) for the effect of each 1 mmol/L increase in maternal glycemia and maternal pre-pregnancy weight status on offspring adiposity measures at birth (n = 1096).

	Macrosomia		LGA	
	*RR*(95% CI)	*P* value	*RR*(95% CI)	*P* value
Model 1^1^				
Maternal FPG	3.74(1.85, 7.60)	0.00	3.01(1.54, 5.88)	0.001
Model 2				
Maternal PPWS (ref: NW)				
UW	0.44(1.00, 1.96)	0.28	0.34(0.08, 1.51)	0.16
OWO	3.14(1.14, 8.66)	0.03	2.29(0.86, 6.07)	1.00
Model 3				
Maternal FPG	3.31(1.62, 6.79)	0.001	2.69(1.35, 5.33)	0.01
Maternal PPWS (ref: NW)				
UW	0.51(0.11, 2.31)	0.38	0.39(0.09, 1.70)	0.21
OWO	2.51(0.89, 7.05)	0.08	1.89(0.70, 5.10)	0.21
Model 4^2^				
FPG*Maternal PPWS	1.14(0.36, 3.64)	0.83	1.19(0.38, 3.74)	0.77

^1^Woman age at delivery, gestational weight gain (GWG), and gestational week were adjusted in all the models. ^2^Model 4: model 3 + maternal FPG*PPWS. FPG: fasting plasma glucose; PPWS: pre-pregnancy weight status; UW: underweight; NW: normal weight; OWO: overweight/obese.

### Effects of maternal FPG level and PPWS on infants’ BMI-z scores in the first year

**Figure 1:**
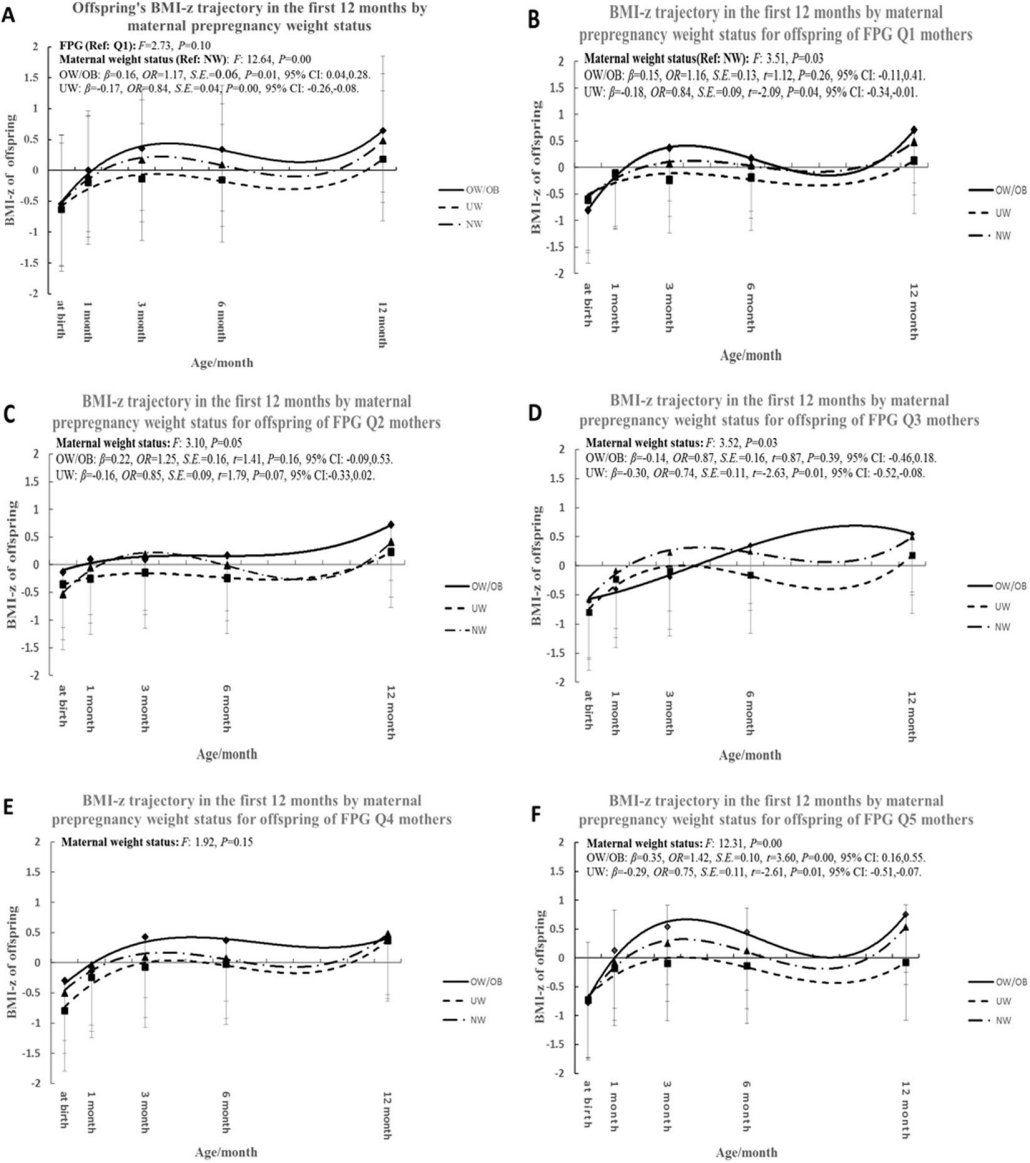
Offspring BMI-z trajectory in the first 12 months of life according to the categories of maternal pre-pregnancy weight status (PPWS) in the overall FPG groups (**A**) and FPG Q1 to Q5 groups (**B** to **F**), separately. All the models were analyzed using the mixed-effects regression method with adjustment for woman age at delivery, gestational weight gain (GWG), gestational week and feeding pattern. FPG categories were as follows: First quintile (Q1), <4.2 mmol/L; second quintile (Q2), 4.2, to <4.4 mmol/L; third quintile (Q3), 4.4, to <4.7 mmol/L; fourth quintile (Q4), 4.7, to <4.9 mmol/L and fifth quintile (Q5), ≥4.9 mmol/L. Q1-Q5: FPG quintile 1–5.

Maternal FPG concentration was further categorized into quintiles, i.e., from Q1 to Q5. With the comparison to infants of a NW mother, infants of underweight (UW) mothers had a decrease of 0.17 in the BMI-z, while infants of an OWO mother showed an increase of 0.16 in the z score (95% CI: −0.26, −0.08; *P* = 0.00 and 95% CI: 0.04, 0.28; *P* = 0.01). No significant effect of the maternal FPG on infant’s BMI-z scores was observed (*P* > 0.05). When performing stratified analysis of maternal FPG, infants of UW mothers had a decrease of 0.18 in the BMI-z for FPQ Q1 mothers compared with infants of NW mothers (95% CI: −0.34, −0.01; *P* = 0.04). Infants of OWO mothers exhibited an increase of 0.15 in the BMI-z but was not significant (95% CI: −0.11, 0.41; *P* = 0.26). Regarding the FPG Q2 group, infants of UW mothers had a decrease of 0.16 in the BMI-z. OWO mothers exhibited an increase of 0.22 in the BMI-z, but neither of the association was significant (95% CI: −0.33, 0.02; *P* = 0.07 and 95% CI: −0.09, 0.53; *P* = 0.16). As for FPG Q3 mothers, infants of UW mothers had a reduction of 0.30 in the BMI-z (95% CI: −0.52, −0.08; *P* = 0.01). Infants of OWO mothers had a decrease of 0.14 in the BMI-z, but was not significant (95% CI: −0.46, 0.18; *P* = 0.39). Regarding the FPG Q4 group, there was no significant association of maternal PPWS with the infants’ BMI-z (*P*  > 0.05). As for the FPG Q5 group, infants of UW mothers had a decrease of 0.29 in the BMI-z (95% CI: −0.51, −0.07; *P* = 0.01). Infants of OWO mothers had an increase of 0.35 in the BMI-z (95% CI: 0.16, 0.55; *P* = 0.00), (Fig. 1).

## Discussion

In this prospective study, the maternal FPG level was significantly and positively associated with macrosomia/LGA at birth. No main effect of the PPWS or interaction association of maternal FPG and PPWS with macrosomia/LGA was observed. The significant interaction between maternal PPWS and gestational FPG was associated with the risk of offspring high BMI-z in the first year.

As far as we are concerned, the FPG level was more powerful in the prediction of birth weight and LGA risk than 1-h and 2-h OGTT glucose levels^17,29,30^. Thus, we used the FPG level in two ways, i.e., FPG value and the five quintile groups, as one of the exposures, in our study.

The present study indicated that maternal hyperglycemia was significantly and positively associated with macrosomia/LGA. This was in keeping with previous work^8,17,23,28^. Zawiejska *et al*.^28^ reported that high FPG level (≥5.1 mmol/l) was related to macrosomia and treatment of insulin and longtime metabolism control, as well as more pregnancy complications. Aris *et al*.^9^ found that gestational FPG was positively associated with birth weight and BMI in infants younger than 3 months, but not older, in the first 3 years. In our study, after further including maternal PPWS, the association remained significant. This was in line with previous research by Zhu *et al*.^19^. They found that among women with GDM, the maternal FPG concentration during pregnancy was significantly and positively associated with offspring birth size and the risk of being OWO, after adjusting for maternal pre-pregnancy BMI. However, Ogonowski *et al*.^31^ found that, other than GDM, pre-pregnancy BMI and GWG were predictive factors of macrosomia among infants in 1,285 Polish women. The different effects of GDM on the outcomes across studies could be due to the difference in the care for GDM. A strict metabolic control as a result of early diagnosis of GDM could produce a better outcome; for example, the reduced risk of high offspring birth weight and thus less macrosomia. However, we could not obtain the data on the GDM care in this study.

The excess adiposity and obesity during pre-pregnancy or early pregnancy are well established modifiable risk factors for developing GDM^32^. Our findings indicated that the significant interaction between maternal PPWS and gestational FPG was associated with the risk of offspring high BMI-z in the first year. When performing stratified analysis of the maternal FPG, we found several interesting phenomena.

First, the correlation between the maternal OWO and increased risk of offspring high BMI-z was only pronounced in individuals in the Q5 FPG group. Pre-pregnancy OWO is a well established risk factor of infant’s overweight^8^ and maternal hyperglycemia was reported to be associated with an increased risk of birth weight and macrosomia which are associated with obesity in the future^33,34^. Inflammation or gene expression modification might interact with maternal obesity and hyperglycemia. Due to the effect of inflammation on the interplay between maternal obesity and GDM, whether inflammation in obese women could lead to GDM or inflammation simply reflects an association of maternal obesity and GDM, are possible influencing factors which are not clear in the present study^35^. The impact of the FPG level at the higher end, without yet having reached the diagnosis standard on infants’ BMI-z in our study, might be mediated through a similar mechanism as that in GDM. Gene expression modification could be an alternative explanation. Some studies hypothesized that maternal hyperglycemia and fuel metabolism in pregnant women might result in fetal exposure to increased amounts of lipid substrates during the third trimester of pregnancy, thereby having long-term effects on the offspring by modifying the phenotypic gene expression continually inducing cell differentiation during intrauterine development^36–38^. We assume this might increase the offspring obesity susceptibility to the long-term impact of maternal OWO and leads to the observed association between maternal OWO and the offspring high BMI-z in the Q5 FPG group. Previous studies, however, reported a contradicting result, as no effect of the interaction between maternal PPWS and FPG on subsequent higher offspring adiposity was found, suggesting that there might be common pathways of inducing a proinflammatory state, excessive fuel substrates and dysregulation in energy balance which may attenuate the independent association between the FPG levels and outcomes^21,36^. Further consideration needs to be given to whether the effect of inflammation or gene expression modification contributes to the interplay role of maternal obesity and gestational glycemia.

Second, in infants born to OWO mothers of the FPG Q3 groups, the BMI-z trajectory showed an upward trend which was contrary to that of the other groups, but the difference was not statistically significant. This might be due to the complexity of various factors, such as breastfeeding, influencing the physical development of children.

The current prospective design and repeated measurements of infants’ anthropometric parameters provided strength to investigate long-term effect of the maternal FPG level during pregnancy on offspring overweight/obesity with adjustment for critical confounders at different stages during the early growth of infants within 1 year of age. Moreover, since, we used standard measurements and trained staffs to obtain data on weight and height from birth to 1 year of age our conclusions can be considered as reliable. Nevertheless, our study had several potential limitations. The lost to follow-up could have reduced the statistical power and introduced selection bias, but the selection bias seemed minimal based on the nonresponse analyses. Also, the use of BMI as the predictor of children’s obesity could be another limitation. The percentage of body fat, for example, assessed using dual energy X-ray absorptiometry (DEXA), is considered as a golden standard for the assessment of obesity. However, several disadvantages, such as high cost, being technically demanding and being subject to the site constrains limit its utilization in large-scale epidemiological studies. Furthermore, children’s exposure to x-ray, albeit at a very low dose, is also of concern to the parents. Accordingly, several anthropometric measurements, e.g., weight for age (WFA), weight for length (WFL), BMI, BMI-z, were used as proxies of childhood obesity^39–41^. To the best of our knowledge, none of the mentioned measurements could be deemed as a perfectly good predictor of childhood obesity. The implication of BMI in childhood obesity in later life has long been controversial as well. However, in contrast to WFA and WFL, the BMI combines weight and length, as well as the age of the children and could be used to assess children everywhere regardless of ethnicity, socioeconomic status and type of feeding^42^. The BMI-z scores were calculated at each age according to the World Health Organization (WHO) standard, which could eliminate the effects of age and gender on the outcome to better assess childhood obesity^39^. Therefore, in the current study, we decided to use BMI-z as the outcome variable.

## Methods

### Ethics Statement

The Ethics Committees of Tongji Medical College, Huazhong University of Science and Technology approved this study. Parents, caretakers, or guardians gave written informed consent on behalf of all the infants involved in the study. All experiments were performed in accordance with relevant guidelines and regulations.

#### Participants

**Figure 2:**
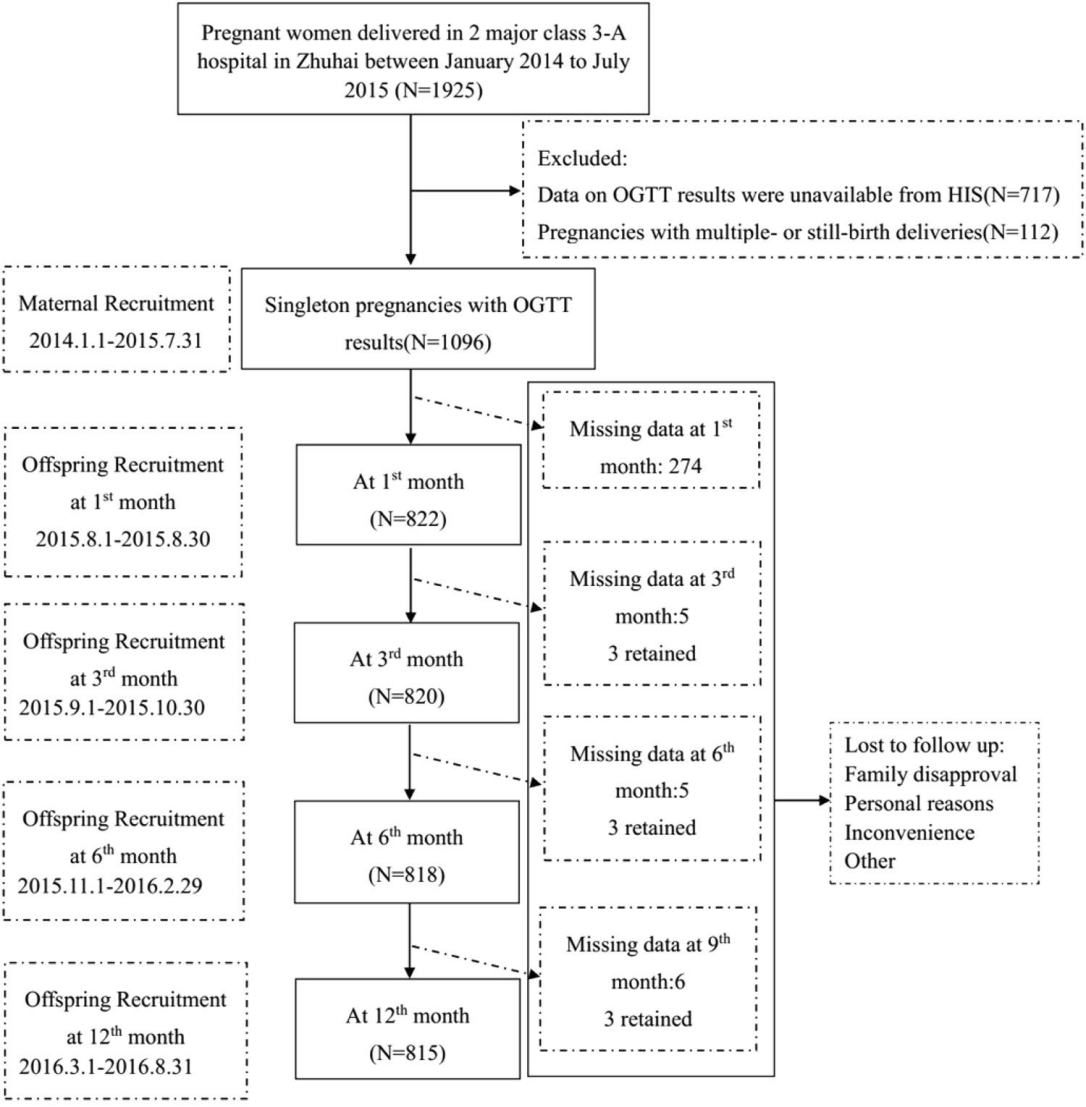
Recruitment and loss to follow up.

The enrolled participants were pregnant women whose age was >18 years with a singleton pregnancy at 12–16 weeks, who were admitted to two major Class A hospitals in Zhuhai city, Guangdong Province, China, between January 2014 and July 2015. The sample size of 600 was estimated by a simple random sampling method with the formula,

$$({\rm{n}}=\frac{{({Z}_{\alpha }\sqrt{{p}_{0}+{p}_{1}}+{Z}_{\beta }\sqrt{{p}_{0}(1-{p}_{0})+{p}_{1}(1-{p}_{1})})}^{2}}{{({p}_{1}-{p}_{0})}^{2}},\alpha =0.05,\beta =0.10),$$

using 20% and 12% as the estimated rate of overweight and obesity at age 1 in the exposure and non-exposure group, respectively, (*p*_*1*_ = 20%, *p*_*0*_ = 12%). Since it is a cohort study, we used 25% as the estimated rate to lost-to-follow up, the sample size at recruitment was 600/(1–0.25) = 800. Those subjects with preexisting diabetes mellitus (DM) and inflammatory or chronic diseases were excluded. All participants were tested for GDM with a 2-h glucose tolerance test (OGTT) performed with a 75 g glucose in solution in the 28^*th*^ gestational week. Women who have had GDM before or had a family history of diabetes were tested during the 12^*th*^ week of gestation. The diagnosis of GDM was confirmed by OGTT using the cut-off values recommended by WHO if fasting glucose was ≥110 mg/dl (6.1 mmol/l) or 2-h glucose was ≥140 mg/dl (7.8 mmol/l)^43,44^. Accurate gestational age was confirmed by an ultrasound examination before 20 weeks of gestation. Of the 1,925 screened women, data on the OGTT results unavailable from Hospital Information System (HIS) (n = 717) as well as pregnancies with multiple- or still-birth deliveries (n = 112) were excluded from the analysis regarding the gestational Apgar score and procedure of delivery. Among the remaining 1,096 pregnant participants who had successful delivery, we included the ones who had at least one offspring anthropometric measure at the 1-month, 3-month, 6-month and 1-year follow-ups into the analysis, and the cohort became 822, 820, 818, 815 at the corresponding follow-up, respectively (Fig. 2).

### Variables

#### Women’s fasting plasma glucose concentration

Fasting plasma glucose (FPG) concentration was measured during the first OGTT during pregnancy, as both continuous and categorical variables were classified into quintiles (Q1-Q5) according to the number of plasma glucose values of the OGTT.

#### Pre-pregnancy weight status (PPWS)

Maternal PPWS was categorized into UW, NW and OWO calculated by self-reported weight and height before pregnancy. The PPWS was another primary exposure using three BMI cutoffs of 18.5, 24 and 28 kg/m^2^ according to the standard of the Working Group on Obesity in China^45^.

#### Gestational weight gain (GWG)

GWG was categorized into three variables, namely adequate GWG, excessive GWG above the adequate GWG and non-excessive GWG. Adequate GWG was classified according to the Chinese maternal pre-pregnancy BMI classification standard and the 2009 IOM GWG recommendations: 12.5–18 kg (BMI < 18.5 kg/m^2^), 11.5–16 kg (BMI: 18.5–23.9 kg/m^2^), 7–11.5 kg (BMI: 24.0–27.9 kg/m^2^), and 5–9 kg (BMI ≥ 28 kg/m^2^)^46,47^.

#### Macrosomia/Large for gestational age (LGA)

Macrosomia/LGA at birth and sex- or age-specific z-score of body Mass Index (BMI) of infants at follow-ups were considered outcome variables. Macrosomia was defined as birth weight ≥4000 g. We classified newborns as LGA when the birth weight, according to the gestational age and sex-specific intergrowth-21^*st*^ curves, was above the 90^*th*^ percentile^48^.

#### Body Mass Index z-score (BMI-z score)

BMI-z score was another outcome variable calculated through the execution of a SAS macro based on the 2006 WHO growth standards from height and weight measured with the participant wearing light clothing or calculate lean weight by minus clothing weight. The length and weight measurements at birth, 3 months, 6 months and 1 year were performed by the trained doctors and nurses. The length and weight at 1 month was extracted from the Hospital Information System (HIS). Infants’ body weight was measured to the 0.05 kg using a Hengxin HCS-20-YE electronic weighing scale and length to the nearest 0.1 cm using a Hengxin HX-II horizontal baby bed twice, and the average were used for the analysis.

#### Feeding pattern

Feeding pattern was recorded as a three-categorical variable, such as exclusive breast feeding (EBF), mixed feeding (MF) and formula feeding (FF). The definition of EBF differed at different times as follows: (1) early initiation of breastfeeding: proportion of children born in the last 24 months who were put to the breast within 1 h of birth; (2) exclusive breastfeeding under 6 months: proportion of infants 0–5 months of age who were fed exclusively breast milk during the previous day; (3) continued breastfeeding at 1 year: proportion of children 12–15 months of age who were fed any breast milk during the previous day. MF was defined as were fed with, but not limited to, breast milk and formula milk, while FF was defined as fed with formula milk and other food but not breast milk^49^.

#### Demographic, family and lifestyle factors

Data on social-demographic characteristics (i.e. sex, age, educational status), and lifestyle factors (i.e. smoking, drinking status, feeding pattern) were collected via structured self-reported questionnaires. Data on gestational DM indicators (i.e. FPG concentration, measured at a 2-h 75 g OGTT after overnight fasting during pregnancy), gestational information (i.e., parity, delivery mode, gestational week) and infants’ weight/length at birth, 1 month (30–59 days), 3 month (90–119 days), 6 month (180–209 days) and 1 year (365–394 days) was recorded in the Hospital Information System (HIS) by the trained doctors and nurses in the delivery room or were measured by the child healthcare doctors when the mothers/infants made their scheduled visits to the hospital with unified measurement tools and approach. We contacted parents by phone and mobile text message 15 days before the due day to remind them of the scheduled visit. When they came back to the hospital, we performed the infants’ body measurements and handed out questionnaires. Educational status was divided into four levels: middle school and under, high school, college, university and above. Delivery mode was categorized into two groups: natural and cesarean delivery.

#### Missing data

Variables of maternal pre-pregnancy BMI, Smoking/drinking during pregnancy, delivery mode had 0.5% missing data, GWG, cars had less than 3% missing data. Due to the small amount of missing data for major variables, we could draw conclusions by our test. Variables of monthly household income had 17.2% missing data, maternal educational level had 20.2% missing data, paternal educational level had 21.4% missing data. Since there was a high proportion of missing data for some exposures, we performed secondary analyses in which we repeated the main analyses after multiple imputation of the missing data. There were no difference between follow-up and total population regarding the baseline characteristics, thus we assumed that the data were missing at random (dependent on values of other non-missing variables), (Table 2).

#### Statistical analysis

The database was established on Epidata 3.1. All analyses were conducted with the SAS 9.3 software (SAS Institute, Cary, NC, USA). Statistical significance level was set at 0.05.

Descriptive characteristics are presented as means ± SD or median (IQR) for continuous variables with normal distribution or skewed distribution, respectively. Categorical variables were presented as percentage and rate. The differences of means on continuous variables such as children’s growth measures and mother’s gestational measures across quintiles of FPG concentration were compared using ANOVA or the Kruskal-Wallis test based on their distribution, and chi-square test was employed to compare the differences of categorical variables, such as educational level, feeding pattern, etc.

Nonresponse analyses were conducted to assess the subjects initially recruited and the remaining subjects at each follow-up. The numbers of non-missing/missing anthropometric data were 822/274, 820/276, 818/278, 815/281 at the 1, 3, 6 and 12 months follow-up, respectively.

Multivariate logistic regression was used to detect the independent and interaction associations of maternal FPG concentrations and PPWS with birth outcomes (i.e. macrosomia and LGA at birth), with confounders, such as woman age, GWG and gestational week adjusted for).

The total samples were first stratified by maternal glucose to assess the impact of the FPG level at the higher end, without yet having reached the diagnosis standard on infants’ BMI-z in our study, and second to examine the interaction between FPG level and PPWS using linear mixed effects (LME) models. These models account for the correlation between repeated measurements and allow for incomplete outcome data^50^. The fitness of the models was examined graphically to assess the normality of the residuals and regression requirements. Time in the models is the age of the child as a continuous variable (months) in each measurement. Cubic-polynomial of time was used to fit the models using the restricted estimated maximum likelihood (REML) method, which considers the loss of the degree of freedom from the unknown fixed effects of the estimated models. Models were also tested for random effects (G matrix) of the intercept and slope and both were included in the models. Unstructured working covariance matrix was chosen for the G matrix^50^. The variable of months corresponding to the BMI-z measurements of each point was chosen as the first level covariate. On the analysis of the second level variables, a significant association of maternal PPWS with infants’ BMI-z was observed in the models. We also found that the adjusted variables of GWG, gestational week and feeding pattern were significantly associated with the infants’ BMI-z and chosen to fit the models. To our knowledge, maternal endocrine disorders occur much easier along with the increase of age, thus we also included the covariate of maternal age in the models. Infants who are exposed to intrauterine hyperglycemic environment, i.e., gestational diabetes mellitus may develop childhood obesity^16^. Therefore, the results were stratified by maternal FPG levels across ages.

### Data Availability Statement

Date are available from the Ethics Committee of Tongji Medical College, Huazhong University of Science and Technology. Data are from the cohort study whose authors may be contacted at 13 Hangkong Road, Department of Woman and Child’s Care and Adolescent Health, School of Public Health, Tongji Medical College, Huazhong University of Science and Technology, Wuhan 430030, Hubei, P.R. China. The contactors could be Jianduan Zhang, jd_zh@hust.edu.cn, or Yilin Huang, yilinh@hust.edu.cn.
